# Upregulation of long noncoding RNA growth arrest-specific 5 mediates pro-inflammatory mechanisms of diabetic wound healing impairment

**DOI:** 10.46439/derma.1.004

**Published:** 2021

**Authors:** Shaquia Idlett-Ali, Kenneth Liechty, Junwang Xu

**Affiliations:** 1 Laboratory for Fetal and Regenerative Biology, Department of Surgery, University of Colorado, Denver-Anschutz Medical Campus and Children’s Hospital of Colorado, Aurora, Colorado 80045, USA; 2 Department of Physiology, College of Medicine, University of Tennessee Health Science Center, Memphis, Tennessee 38163, USA

**Keywords:** Diabetes, lncRNA GAS5, Wound healing, Macrophage polarization

## Abstract

Unresolved inflammatory processes contribute to impaired healing in diabetic wounds, with increasing evidence implicating persistent pro-inflammatory macrophage polarization as a driver of chronic inflammation and delayed wound closure. Previous investigations aimed to uncover the role of regulatory RNAs in macrophage polarization and to understand how aberrant expression patterns contribute to wound healing impairment, with the goal of identifying novel therapeutic targets for promoting normal wound healing progression. In the Journal of Investigative Dermatology, Hu et al. reveal a role of the tumor suppressor, long noncoding RNA (lncRNA) Growth Arrest-Specific 5 (GAS5), in regulating macrophage polarization. Of note, their findings suggest that hyperglycemia induces overexpression of GAS5 which subsequently results in a greater production of the pro-inflammatory macrophage phenotype. Knockdown of GAS5 in diabetic wounds normalized healing time, highlighting the potential therapeutic value of targeting GAS5 for enhanced wound healing progression.

## Introduction

Complications arising from impaired healing of foot ulcers commonly result in non-traumatic lower extremity amputation, with diabetic patients comprising 50% of those undergoing amputation [1]. Though the large burden of diabetic foot disease is well described [2,3], there remains a dearth of effective therapies to treat impaired wound healing and improve patient outcomes. Chronic inflammation has been identified as a key feature of diabetic wounds, with hyperglycemia and persistent pro-inflammatory macrophage (M1) polarization limiting progression to pro-regenerative stages of wound healing. The wound healing cascade is a complex process that can be modified at each stage by regulatory RNAs, including long noncoding RNAs (lncRNAs). LncRNAs enact their regulatory function by binding to DNA, RNA, or protein; suggestive of an ability to modify genetic targets at multiple levels [4,5]. In the recent publication by Hu et al. in the Journal of Investigative Dermatology, the role of a known tumor suppressor—lncRNA Growth Arrest-Specific 5 (GAS5) [6], in diabetic wound healing was investigated [7]. Here we describe their key findings, with an emphasis on the implications in macrophage polarization.

## Hyperglycemia Induces Higher GAS5 Expression in Macrophages and Fibroblasts

Elevated blood glucose with subsequent increases in inflammatory mediators and cytokines generates a microenvironment conducive to prolonged inflammation with limited resolution and repair of wounds. Hu et al. revealed that GAS5 expression was elevated in human diabetic skin and the dermal skin of diabetic mouse models [7]. Additionally, the article reported that wound macrophages isolated from diabetic mice expressed elevated levels of GAS5, and hyperglycemic environment induced GAS5 expression patterns in RAW macrophages similar to the M1-like phenotype. Hu et al. also demonstrated that non-diabetic fibroblasts transition to diabetic-like fibroblast expression levels of GAS5 in a hyperglycemic environment [7]. Combined these results suggest a mechanism of hyperglycemia-mediated chronic inflammation that involves the dysregulation of GAS5 expression, leading to elevated production of pro-inflammatory (M1-like) macrophages and abnormal fibroblasts.

## GAS5-Mediated Healing Impairment is Due to Preferential Pro-Inflammatory Macrophage Polarization

Prolonged healing times in diabetic wounds have been associated with the inability to progress from the pro-inflammatory to pro-regenerative stages of wound healing. This is frequently linked with persistent pro-inflammatory macrophage (M1) polarization. Compared to pro-regenerative macrophages (M2), elevated gene expression of inducible nitric oxide synthase (iNOS), interleukin-1 beta (IL-1β), and tumor necrosis factor-alpha (TNFα) is observed in M1 macrophages [8]. Hu et al. found that overexpression of GAS5 preferentially induced an M1-like macrophage phenotype, indicated by increased expression of these M1 marker genes and elevated convergence of M1 surface markers, CD11b and CD86 [7].

Factors contributing to GAS5-mediated wound healing impairment could be related to isolated or combinatory induction of M1 polarization and inhibition of M2 polarization. Signal Transducer and Activator of Transcription 1 (STAT1) is a known inducer of M1 macrophages [9,10] and Hu et al. found that GAS5 induction of M1-like macrophages was STAT1-dependent [7]; identifying a mechanism of GAS5-mediated healing impairment. Previous evidence suggests that overexpression of GAS5 also inhibits M2 polarization [11,12]. Mechanisms of GAS5-mediated inhibition of M2 polarization have been identified as enhanced phosphatase and tensin homolog (PTEN) expression [11] and silencing of the C-C motif chemokine ligand 1 (CCL1) gene—a chemokine essential for prolonging the life of M2b macrophages [12]. Knockdown of GAS5 normalized healing progression and decreased the wound closure time to 14 days, mirroring the non-diabetic controls. The epithelial gap was also reduced with GAS5 knockdown, which may result from the known effect of GAS5 on the attenuation of fibroblast activation and proliferation [13,14]. Interestingly, wound healing promoted by topical application of mevastatin has been associated with increased expression of GAS5 and subsequent inhibition of c-Myc, a marker of impaired wound healing [15]. While GAS5-mediated c-Myc suppression contributes to wound healing, in parallel pathways mevastatin also reduced wound cortisol levels, promoting epithelialization and angiogenesis [15], key processes in wound healing. GAS5 may have varying effects in different tissues [16–19], so additional studies will be critical to further delineate its mechanism of action in diabetic skin.

Additional work from this research group identified a differential expression pattern of microRNA (miRNA)-21 during diabetic wound healing, which was linked to persistent M1 macrophage polarization [8]. MiRNA-21 has been identified as a target of GAS5, with high GAS5 expression correlating with low miRNA-21 levels in cancer cells [20,21]. Upregulation of GAS5 may contribute to “subthreshold” levels of miRNA-21, leading to insufficient PTEN and programmed cell death 4 (PDCD4) inhibition, with subsequent limitation of the M2 macrophage transition [22]. Overall, this research suggests an interplay between GAS5 and miRNA-21 expression in regulating macrophage polarization and wound healing progression.

## Conclusion

Hyperglycemia-associated upregulation of lncRNA GAS5 may be a key, modifiable factor contributing to impaired wound healing. Therapies targeting this regulatory RNA could be beneficial for mitigating complications associated with diabetic foot ulcers.
